# The Adsorption of Dextranase onto Mg/Fe-Layered Double Hydroxide: Insight into the Immobilization

**DOI:** 10.3390/nano8030173

**Published:** 2018-03-19

**Authors:** Yi Ding, Le Liu, Yaowei Fang, Xu Zhang, Mingsheng Lyu, Shujun Wang

**Affiliations:** 1 College of Marine Life and Fisheries, Huaihai Institute of Technology, Lianyungang 222005, China; leonardoding@sina.com (Y.D.); liulezz3@163.com (L.L.); foroei@163.com (Y.F.); 2 Jiangsu Institute of Marine Resources Development, Huaihai Institute of Technology, Lianyungang 222005, China; 3 Co-Innovation Center of Jiangsu Marine Bio-industry Technology, Huaihai Institute of Technology, Lianyungang 222005, China; 4 Verschuren Centre for Sustainability in Energy & the Environment, Cape Breton University, Sydney, NS B1P 6L2, Canada; xu_zhang@cbu.ca

**Keywords:** layered double hydroxide, dextranase, adsorption, amino acids

## Abstract

We report the adsorption of dextranase on a Mg/Fe-layered double hydroxide (Mg/Fe-LDH). We focused the effects of different buffers, pH, and amino acids. The Mg/Fe-LDH was synthesized, and adsorption experiments were performed to investigate the effects. The maximum adsorption occurred in pH 7.0 4-(2-hydroxyethyl)-1-piperazineethanesulfonic acid (HEPES) buffer, and the maximum dextranase adsorption uptake was 1.38 mg/g (416.67 U/mg); histidine and phenylalanine could affect the adsorption. A histidine tag could be added to the protein to increase the adsorption significantly. The performance features and mechanism were investigated with X-ray diffraction patterns (XRD) and Fourier transform infrared spectra (FTIR). The protein could affect the crystal structure of LDH, and the enzyme was adsorbed on the LDH surface. The main interactions between the protein and LDH were electrostatic and hydrophobic. Histidine and phenylalanine could significantly affect the adsorption. The hexagonal morphology of LDH was not affected after adsorption.

## 1. Introduction

The immobilization of biomolecules, such as nucleotides or proteins, has been widely studied. Biomolecules assembled onto or within nanoscale inorganic materials are novel nano-biohybrid materials, and constitute a new generation of materials at the interface of biology and materials science [1]. Bioinorganic systems to immobilize enzymes are an important step towards developing biosensors and biocatalysts [2,3]. Biohybrids of enzymes and inorganic solids can protect the enzymes from decomposition and denaturation. This results in a high enzyme density, high enzymatic activity, long-term stability, and good substrate accessibility.

Layered double hydroxides (LDHs) are also known as anionic clay or hydrotalcite [4], and are inorganic nanomaterials with positively charged brucite-like layers of mixed metal hydroxides defined by the general formula [M^II^_1−X_M^III^_X_(OH)_2_]^X+^ [(A_X/n_)^n−^·mH_2_O] [5]. LDHs exist in nature, and can be prepared artificially. LDHs with different divalent and/or trivalent metal ions have many variations and combinations, as well as different properties [6]. There have been many reports about Mg/Al-LDH [7,8], but Mg/Fe-LDH has rarely been reported. LDHs have attracted considerable attention as host structures in catalysis, separation technology, nanocomposites, materials engineering, and medical science [9]. These structures have good biocompatibility, low toxicity, good anionic exchange capacity, and favorable adsorption behavior. These inorganic nanomaterials can also immobilize enzymes [10,11]. Several reports have shown that the immobilization of LDHs and enzymes can preserve the enzymes’ activities and properties during charge transport [12,13]. There are some reports about intercalation methods for organic molecules into LDHs, and the kinetic study of the exchange of anions in LDHs has been also reported [14,15].

Dextranases (EC 3.2.1.11) [16] can hydrolyze the α-1,6 glucosidic bond contained in dextran [17,18]. They have been isolated from many microorganisms, such as fungi, yeasts, and bacteria, and can be classified according to their mechanisms of action [19,20,21]. Dextranase can remove dextran from sugarcane in the sugar industry and can be used in oral medicine to prevent dental caries [16,22]. In our laboratory, dextranase was isolated from marine *Arthrobacter oxydans* KQ11 (ncbi-n: JX481352.1) collected from mud local to Lianyungang, China (the Yellow Sea) [23]. Dextranase from *A. oxydans* KQ11 has some unique properties, including low-action temperature, short production time, and good alkaline stability. However, the stability of dextranase from *A. oxydans* KQ11 must still be improved [24]. There are several studies on the immobilization of dextranase and materials, such as bentonite (montmorillonite), hydroxyapatite, Streamline DEAE, and Euperigit C [25,26]. Such *A. oxydans* KQ11 dextranase immobilization could stabilize and preserve it in alkaline environments.

In this study, dextranase from *A. oxydans* KQ11 was selected as a model enzyme, and was adsorbed onto Mg/Fe-LDH materials. We immobilized the enzyme via a co-precipitation process to synthesize a new biohybrid of Mg/Fe-LDH/dextranase, and characterized the biohybrid. We then studied the effects of adsorption via amino acids and phosphate buffer to explore the mechanism of adsorption.

## 2. Results

### 2.1. Adsorption Study

#### 2.1.1. Optimum Buffers for Adsorption

**Figure 1:**
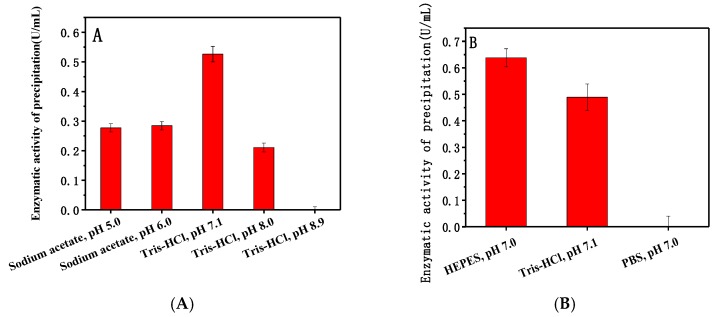
Effect of different pH buffers on the adsorption of dextranase onto Mg/Fe-LDH (**A**) pH 5.0–8.9; (**B**) pH 7.0.

Buffers used here included 50 mM acetic acid sodium acetate buffers (pH 5.0 and 6.0) and 50 mM Tris-HCl buffers (pH 7.1, 8.0, and 8.9). Figure 1A shows that the enzymatic activity of the precipitate was highest in pH 7.1 Tris-HCl. Therefore, three buffers near pH 7.0 were used for further research. The enzymatic activity of the product was highest with pH 7.0 HEPES (Figure 1B). The enzymatic activity can estimate the amount of dextranase adsorbed onto Mg/Fe-LDH. In general, the maximum amount of immobilized enzyme was with pH 7.0 HEPES.

#### 2.1.2. Adsorption Isotherm

**Figure 2:**
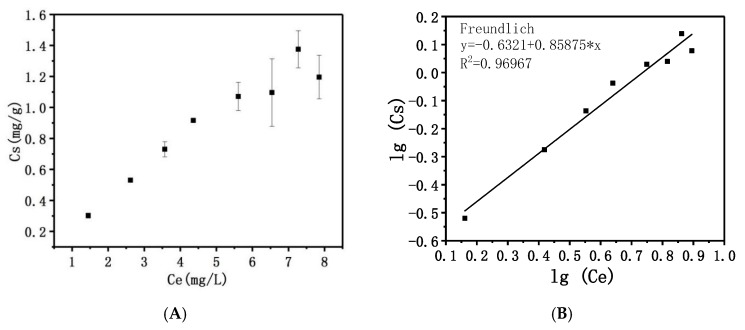
Adsorption isotherm of dextranase onto Mg/Fe-LDH (**A**) and modeling according to Freundlich equation (**B**).

To determine the adsorption capacity of Mg/Fe-LDH, an adsorption isotherm was established using Mg/Fe-LDH suspensions (4 mg·mL^−1^). Figure 2A showed the adsorption isotherm of dextranase from *A. oxydans* KQ11 onto Mg/Fe-LDH. The specific activity (SA, U/mg) of dextranase was determined as 416.67 U/mg. According to the classification of the solute adsorption isotherms reported by Giles et al. [27], this is not completely L-type.

#### 2.1.3. Adsorption Mechanism

**Figure 3:**
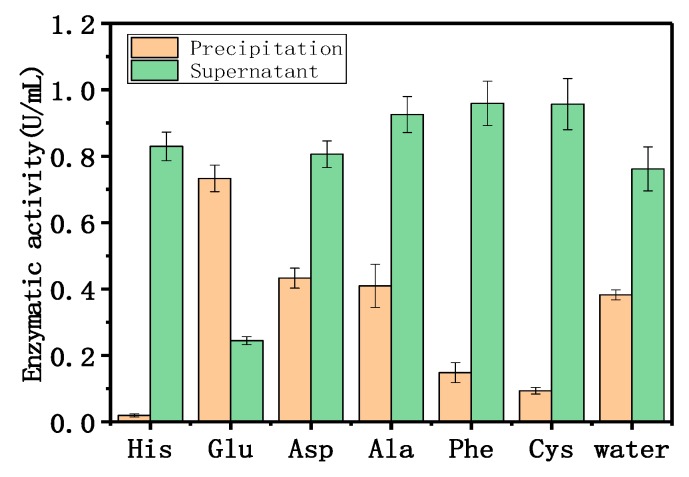
Effect of different amino acids on adsorption.

The adsorption mechanism was determined using different amino acids and phosphate anions. These species replaced the enzyme adsorbed onto the LDH and eluted the immobilized enzyme. Figure 3 shows the effect of 50 mM amino acids on the adsorption of dextranase onto Mg/Fe-LDH. Histidine, phenylalanine, and cysteine could obviously affect the adsorption because of the lower enzymatic activities of the precipitates and the higher enzymatic activities in the supernatant. Aspartate and alanine did not affect the adsorption; glutamate increased the adsorption. Histidine and cysteine are hydrophilic amino acids, and phenylalanine is a hydrophobic amino acid. 

**Figure 4:**
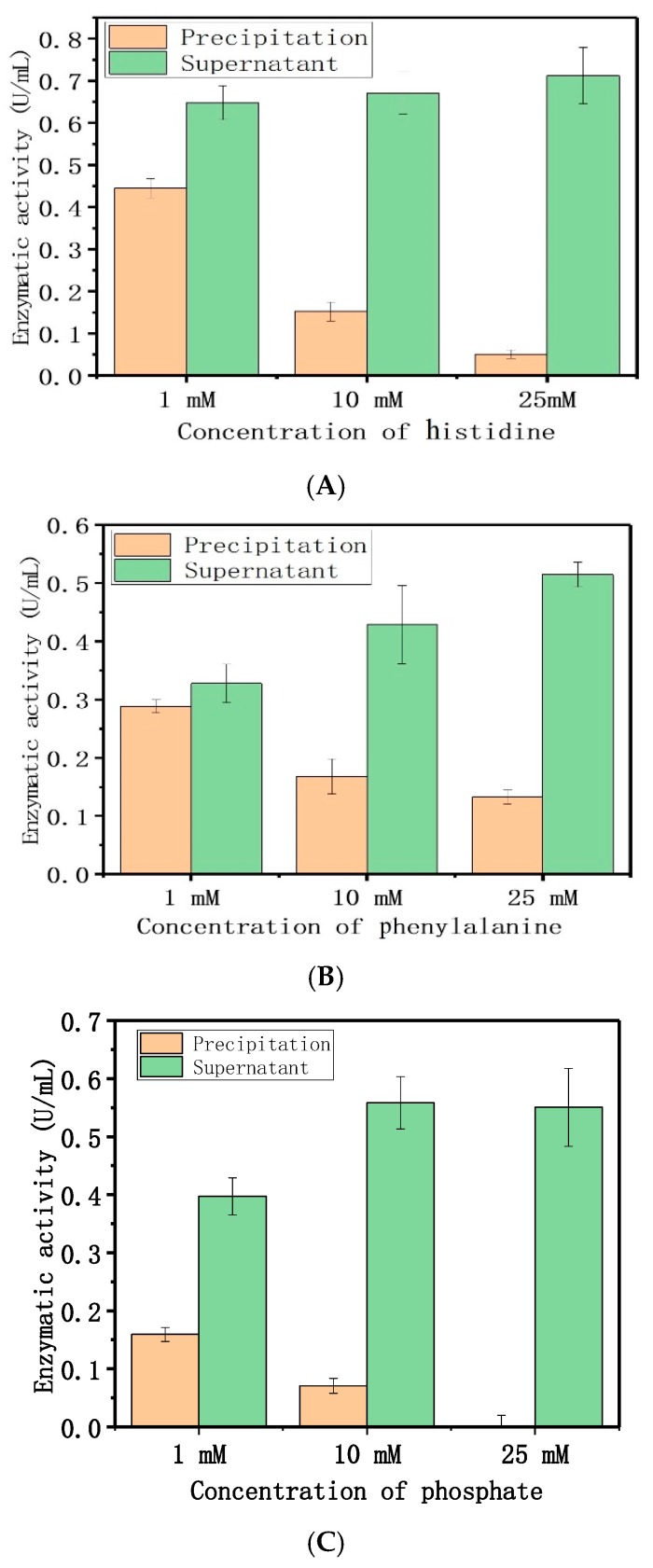
Effect of different concentrations of amino acids and phosphate on adsorption: (**A**) histidine, (**B**) phenylalanine, (**C**) phosphate.

**Figure 5:**
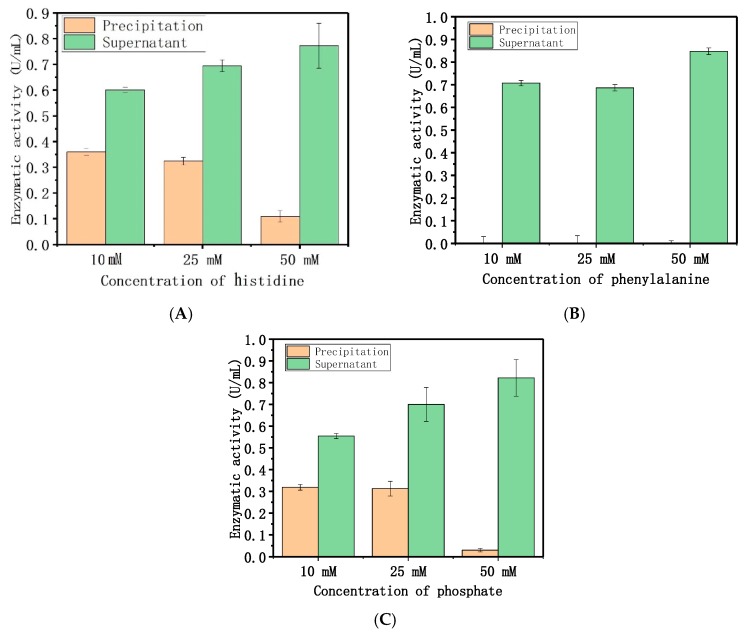
Effect of different concentrations of amino acids and phosphate on the elution of dextranase: (**A**) histidine, (**B**) phenylalanine, (**C**) phosphate.

The results indicated that the electrostatic and hydrophobic forces were dominant, and drove the adsorption between dextranase and LDH. The PBS (Phosphate Buffered Saline) buffer affected the adsorption, which further supports our speculation. Therefore, different concentrations (1, 10, and 25 mM) of histidine, phenylalanine, and disodium phosphate were used for a similar study (Figure 4). These results indicate that the amount of immobilized enzyme gradually decreased with increasing amino acid and phosphate contents. Figure 5 showed that different concentrations (10, 25, and 50 mM) of histidine, phenylalanine, and disodium phosphate were used to elute the adsorption. The elution increased the adsorption concurrent with the increase in histidine and phosphate concentrations. The concentrations of phenylalanine tested here could also completely remove the enzyme. Meanwhile, the other two cationic amino acids (arginine and lysine) were used for the same procedure, to show the electrostatic interactions between the protein and LDH (Figures S3 and S4).

**Figure 6:**
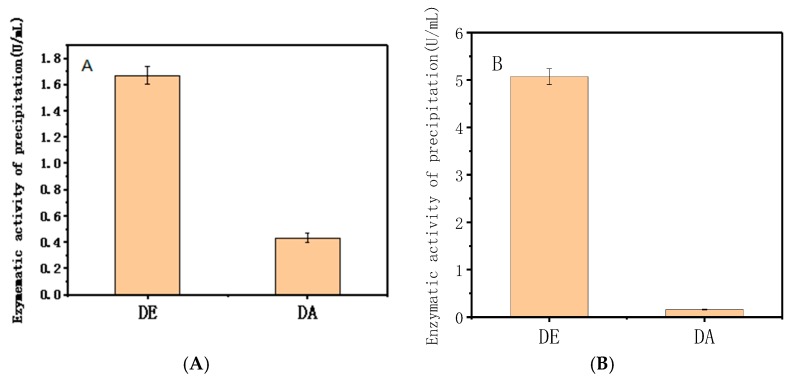
Effect of six-histidine tags in the protein sequence on adsorption (**A**) detected based on same enzyme active, (**B**) detected based on same concentration of protein of enzyme.

Figure 6A shows that at the same enzymatic activity, DE has much more adsorption capacity onto Mg/Fe-LDH than DA. Similar results occur at the same protein concentration (Figure 6B). Therefore, the six-histidine tags in the protein sequence could significantly affect the adsorption of dextranase onto Mg/Fe-LDH.

### 2.2. Characterization

**Figure 7:**
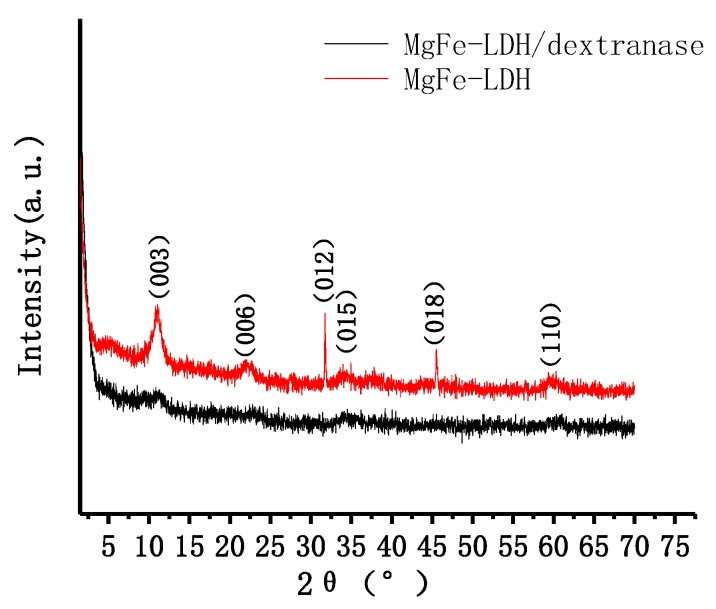
XRD patterns of Mg/Fe-LDH and Mg/Fe-LDH/dextranase.

**Figure 8:**
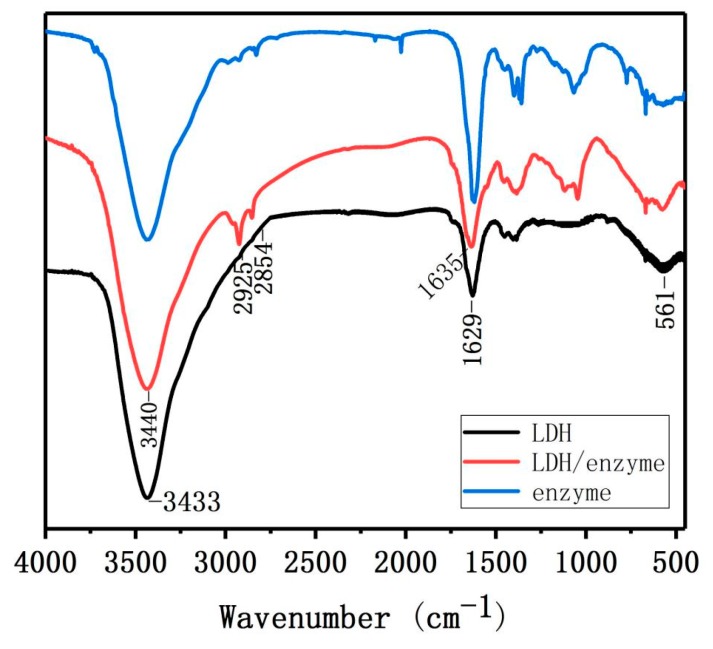
FTIR patterns for Mg/Fe-LDH, Mg/Fe-LDH/dextranase, and dextranase.

**Figure 9:**
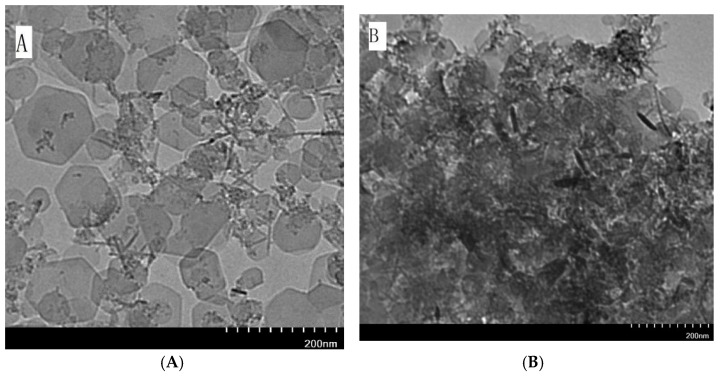
TEM images of (**A**) MgFe-LDH and (**B**) MgFe-LDH/dextranase.

Powder X-ray diffraction was used to investigate the layer spacing and crystallinity of LDH. The XRD pattern of Mg/Fe-LDH (Figure 7) displays the characteristic diffraction peaks (003), (006), (012), (015), (018), and (110). The XRD pattern of biohybrid of Mg/Fe-LDH/dextranase also displays characteristic diffraction peaks (003), (006), (015), and (110), but the peak broadening and increased FWHM (estimated by MDI jada 5.0) indicated a reduced crystallinity of the biohybrid. Figure 8 details the FTIR spectra for the Mg/Fe-LDH, biohybrid of Mg/Fe-LDH/dextranase, and the free dextranase. The surface morphology of Mg/Fe-LDH before and after immobilization was characterized via TEM images. Figure 9 (panel A) represents the nanograph of Mg/Fe-LDH.

## 3. Discussion

Considering the isoelectric point (pI 4.6) of dextranase, this enzyme should be adsorbed favorably onto the positively charged layers of LDH with electrostatic attraction over pH 7.0. Indeed, in some buffer conditions at pH 7.0, LDH showed better adsorption capacity for this enzyme. But the result showed that LDH lost adsorption capacity in pH 7.0 PBS. PBS is a common physiological buffer and that can be widely used in bioapplications. Unfortunately, pH 7.0 PBS could not be used for the adsorption because of the existence of phosphate (as demonstrated by the following experiment). Instead, we used two more kinds of buffers (barbitone sodium pH 7.0 and borate saline pH 7.4) with pH values of about 7.0 (Figure S1). Those two buffer conditions also showed good adsorption capacity.

Gondim et al. reported the maximum adsorption of immunoglobulin G (IgG) and human serum albumin (HAS) onto Mg/Al-LDH in the pH 7.2 Tris-HCl buffer and pH 4.8 acetate buffer, respectively [28]. The results suggested that different proteins are optimal in different buffers and at different pH values. The type and ratio of divalent or trivalent metals in LDH can also affect the adsorption of proteins. The isoelectric point of the proteins might underlie the effect of pH on the buffer.

The adsorption isotherm can be divided into two parts. A net increase of the amount of immobilized enzyme was observed at low equilibrium concentrations (Ce below 4.5 mg/mL). Beyond the Ce value, there was a plateau with an adsorption capacity of 1.38 mg/g. This suggests that the adsorption proceeds in two steps: linear progression of adsorption followed by saturation of the adsorption sites’ mineral structure.

Two models [12] have been developed to describe the adsorption isotherm. The experimental data was analyzed according to Langmuir (Equation (1)) and Freundlich (Equation (2)) equations (Figure 2 and Figure S2):

(1) Ce/Cs=Ce/Cm+1/(Cm×L)

(2) lgCs=lgKf+(1/n)×lgCe

Here, *Cm* and *Kf* are constants representing the adsorption capacity. *L* and *n* are constants that estimate the affinity between the adsorbate and adsorbent. The correlation coefficients R^2^ showed that the adsorption isotherm was fitted to the Freundlich equation, and not fitted to the Langmuir equation. This suggests that the adsorption might occur over multiple layers. This suggested that the adsorption is not due to one interaction force.

In general, histidine, phenylalanine, and phosphate obviously affected the adsorption of dextranase onto Mg/Fe-LDH. Arginine and lysine could also affect the adsorption in varying degrees. This means that the main adsorption forces between dextranase and layers of Mg/Fe-LDH are electrostatic or hydrophobic forces. The amino acid sequence of dextranase from *A. oxydans* KQ11 (total 641 AAs) is listed in the Supplementary Materials, which shows that there are 319 hydrophobic sites and 63 cationic sites in this sequence (Table S1). Some of the hydrophobic sites and cationic sites in the protein sequence might be adsorption sites onto Mg/Fe-LDH.

We engineered *E. coli* BL21(DE3) [29] KQ11-Cold III to produce dextranase. The main difference between the dextranase (DA) from the wild type *A. oxydans* KQ11, and the mutant (DE) from *E. coli* BL21(DE3) KQ11-Cold III is six additional histidine tags in the protein sequence of DE. The 6×His-tagged DE has been purified by affinity chromatography column Ni-NTA Resin [30] (5 mL, purchased from Thermo). DE was adsorbed onto Mg/Fe-LDH to verify the histidines’ effect on the adsorption and compare it to DA.

The amino acid sequence is unique for the acidic, alkaline, or polar amino acids, and this could affect the adsorption. However, the protein will form a tertiary structure that modulates the bioactivity. The histidine tags were expressed outside of the protein, and this obviously affects the adsorption. The isoelectric point is governed by the amino acid sequence, but the adsorption might be affected by how many acidic, alkaline, or polar amino acids were exposed to the surface when the tertiary structure of the protein formed.

The enzyme is adsorbed on the surface of LDH. According to the Bragg’s Law, the average interlayer distance of the materials can be calculated: d(Mg/Fe-LDH) = 7.9934 Å; d(Mg/Fe-LDH/dextranase) = 8.1551 Å [31]. The interlayer distance of the LDH material did not markedly change after being adsorbed by dextranase. These results confirmed the feasibility of LDH as an immobilizing agent.

In the Mg/Fe-LDH spectrum, the 3433 cm^−1^ or 3440 cm^−1^ peaks belong to the stretching bands of O–H and N–H. The C–H, C–O–C, and C–O stretching bands were at 2925 cm^−1^ and 2854 cm^−1^. The band at 1635 cm^−1^ belongs to the amide groups of amino acids. This confirmed that dextranase was adsorbed on the surface of the LDH. The adsorption peaks from 1400–1500 cm^−1^ and the one below 800 cm^−1^ belong to the vibration characteristic peaks of bonds Mg–O and Fe–O [32,33]. The results suggested that the dextranase from *A. oxydans* KQ11 was definitely adsorbed onto the surface of LDH material through a co-precipitation method [33]. All of the activity data suggested that the enzyme was not denatured.

The TEM images showed typical hexagonal particles of LDH that were smaller than 200 nm which could result in the formation of clusters. The modified aggregation of LDH was seen in Panel B after immobilization of dextranase, and is due to adsorption. The adsorption process seemed to obstruct the crystalline growth of LDH slightly, and the hexagonal particles were still obvious. The results also indicated that the adsorption was on the surface of the LDH crystallites.

## 4. Materials and Methods

### 4.1. Starting Materials

Dextran-20000 was purchased from Wako Japan. The MgCl_2_·6H_2_O, FeCl_3_·6H_2_O, NaOH, and 4-(2-hydroxyethyl)-1-piperazineethanesulfonic acid (HEPES) were purchased from Sigma Aldrich. All other reagents were of analytical grade, and all solutions were prepared in deionized distilled water.

### 4.2. Preparation of Dextranase

We prepared and purified dextranase from marine *A. oxydans* KQ11, as described [23]. The marine *A. oxydans* KQ11 was cultivated in medium containing 0.4% dextran-20000, 0.4% NaCl, 0.75% soy meal, 0.75% cassava starch, 0.5% wheat bran, and 0.04% MgSO_4_ (pH 7.5). This was fermented aerobically at 30 °C and 150 rpm for 30 h. The fermentation broth was filtered through diatomaceous earth, and the supernatant was filtered through an ultrafiltration membrane. The processed liquid was freeze-dried using a vacuum freezer to create a crude enzyme powder; this was then stored at −20 °C.

The crude enzyme was purified with ammonium sulfate fractionation [34,35]. Briefly, the powdered enzyme was dissolved at 2.0 g per 100 mL distilled deionized water. To remove the small molecular impurities, the enzyme solution was filtered and concentrated through Merck Millipore Labscale tangential flow ultrafiltration system. The concentrated enzyme solution was then added into (NH_4_)_2_SO_4_ (43.6 g/100 mL) slowly on a magnetic stirrer at room temperature until thoroughly dissolved. This was then held at 4 °C for 4 h. The solution was centrifuged at 12,000 rpm for 20 min. The precipitation was collected and resuspended by 5 mM HEPES pH 7.0 (15 mL). The enzyme solution was poured into a dialysis bag to remove the inorganic salts. This dialysis bag was then placed in 5 mM HEPES pH 7.0 (500 mL) and kept at 4 °C for 12 h, and then stored at 4 °C.

### 4.3. Dextranase Activity Assay

The dextranase activity was measured via the Miller method [17,23]. Briefly, 50 μL enzyme solution was added into 150 μL 3% dextran-20000 (50 mM acetate buffer, pH 5.5) [36]. The enzyme solution was not added to the control group. The mixture was incubated at 45 °C for 15 min. Then, 200 μL DNS (3,5-dinitrosalicylic acid) was added to the experimental and control groups to stop the reaction; 50 μL enzyme solution was added to the control group [37]. The mixtures were then boiled for 5 min, and 3 mL pure water was added into the mixture after cooling. The mixtures’ absorbance at 540 nm was measured with a microplate reader. The amount of enzyme required to release 1 μmoL of maltose per minute is an activity unit (U). Enzymatic activities and protein assays were acquired by absorbance values using Thermo MULTISKAN GO microplate reader.

### 4.4. Synthesis of Mg/Fe-LDH

Preparation methods used for nano-layered double hydroxides include co-precipitation [38], induced hydrolysis [39], and urea synthesis [40]. The co-precipitation method is one of the most common methods for preparing LDH in the laboratory, and produces LDH precipitates under alkaline conditions. Briefly, 22.5 mL 0.1 M MgCl_2_, 7.5 mL 0.1 M FeCl_3_, and 14 mL distilled deionized water were added into a three-necked flask under magnetic stirring at 300 rpm for 30 min. The stirring speed was then changed to 500 rpm, and 6 mL 1 M NaOH was added immediately. This was stirred for 30 more minutes. The reaction was performed under an N_2_ atmosphere to avoid the effects of CO_2_ and O_2_. The suspension was then centrifuged at 5000 rpm for 10 min. The precipitate was resuspended with deionized water and centrifuged again to wash away excess NaOH; this was repeated three times. Finally, the suspension was resuspended with ultrasound for 10 min. The concentration of the resulting product of Mg/Fe-LDH suspension was 4 mg/mL, and the ratio of Mg^2+^ to Fe^3+^ was 3:1.

### 4.5. Adsorption Studies

#### 4.5.1. The Synthesis of a Mg/Fe-LDH/Dextranase Biohybrid via Co-Precipitation

Here, 100 μL of buffer was added into 100 μL Mg/Fe-LDH suspension (4 mg/mL). Next, 100 μL dextranase solution was added to the diluted LDH suspensions. The system was mixed at 25 °C at 180 rpm for 30 min. The mixture was then centrifuged at 12,000 rpm for 5 min to collect the precipitate. The resulting biohybrid material was stored at 4 °C and denoted Mg/Fe-LDH/dextranase.

#### 4.5.2. Determination and Quantitation of Adsorption

The precipitation of LDH and enzyme was resuspended with 100 μL HEPES buffer (50 mM, pH 7.0). The reduced sugar was measured via the Miller method to calculate the enzymatic activity of the suspension. A small amount of supernatant remained in the precipitate. The exact volume was estimated with pipettes. The enzymatic activity of the residual supernatant in the precipitate was measured by diluting the same amount of supernatant with 100 μL HEPES buffer (50 mM, pH 7.0) following the same process described above. The enzymatic activity of the precipitate is the suspension minus the residual supernatant. The LDH suspension can be replaced with deionized water as a control group.

#### 4.5.3. Optimum Buffer for Adsorption

To determine the optimum conditions for buffer adsorption, different buffers with different pH values were used to dilute the Mg/Fe-LDH and create buffered adsorption conditions. The diluted Mg/Fe-LDH was used to adsorb dextranase from *A. oxydans* KQ11, and the enzymatic activities of the precipitate were measured. The highest enzymatic activity helped define the ideal buffer conditions for adsorption.

#### 4.5.4. Adsorption Isotherm

First, the 100 μL of optimum buffer was added to 100 μL Mg/Fe-LDH suspensions (4 mg/mL). Next, added 25 μL, 50 μL, 75 μL, 100 μL, 150 μL, 200 μL, 250 μL, and 300 μL dextranase solution were added to the diluted LDH suspensions. This followed the same procedure as described above to measure the total enzymatic activities (Ap, U) for the precipitate. We then measured the activity (U/mL) of the enzyme solution, and the protein concentration of the enzyme solution (mg/mL) was quantified using a BCA assay (Colaber). The specific enzymatic activity (SA, U/mg) and the equilibrium enzyme concentration (me, mg) were calculated.

The adsorption isotherm can be obtained by plotting the amount of immobilized dextranase per gram of LDH solid (Cs, mg/g) vs the equilibrium concentration of dextranase (*Ce*, mg/L), where

$$m_{s}=\frac{A_{p}}{SA},\;Cs=\frac{m_{s}}{m_{L}},\;Ce=\frac{m_{e}}{V}$$

Here, *m_s_* (mg) is the amount of immobilized dextranase; *m_L_* (g) is the amount of LDH; and *V* (L) is the total volume of the suspensions [12].

#### 4.5.5. Adsorption Mechanism

The adsorption mechanism was explored via different amino acids and phosphate anions. Two acidic amino acids (aspartic acid and glutamic acid), one basic amino acid (histidine), one nonpolar amino acid (alanine), one polar amino acid (cysteine), and one aromatic amino acid (phenylalanine) were chosen. Briefly, 100 μL HEPES buffer (50 mM, pH 7.0) was added to 100 μL Mg/Fe-LDH suspensions (4 mg/mL). These were mixed with different concentrations of amino acids and disodium hydrogen phosphate, 100 μL, along with the diluted LDH suspensions for 30 min.

Next, we added 100 μL dextranase, and the system was mixed at 25 °C and 180 rpm for 30 min. The mixture was then centrifuged at 12,000 rpm for 5 min to collect the precipitate. The product was resuspended with 50 μL 50 mM HEPES pH 7.0. We then measured the enzymatic activities of the suspensions and the supernatants. A distilled water control replaced the amino acids and the phosphate buffer. A higher concentration of amino acids and phosphate were used to elute the adsorption. Briefly, 100 μL amino acids and disodium hydrogen phosphate (10, 25, and 50 mM) were added to the adsorption systems afterwards. The enzymatic activities of the supernatant and the precipitate were measured.

### 4.6. Chemical Physical Characteristics

The powders of pure Mg/Fe-LDH and biohybrid of Mg/Fe-LDH/dextranase were prepared and dried in a vacuum oven at 30 °C. Powder X-ray diffraction patterns (XRD) were recorded on a PANalytical X’Pert Powder diffractometer using Cu Kα radiation (λ = 1.540598 Å) at 40 kV and 40 mA. The patterns were recorded over 1.57–0.0° 2θ range in steps of 0.0066° 2θ a measurement time of 13.77 s per step. Fourier transform infrared spectra (FTIR) were recorded in transmission mode using a Bruker Tensor II FTIR spectrophotometer from 4000–400 cm^−1^. The dispersions of Mg/Fe-LDH and Mg/Fe-LDH/dextranase were observed with a Hitachi TEM system H7000 with an accelerating voltage of 80 kV.

## 5. Conclusions

A novel biohybrid material was synthesized by immobilizing dextranase from marine *A. oxydans* KQ11 onto Mg/Fe-LDH via a co-precipitation method. The Mg/Fe-LDH showed better adsorption capacity at a pH value of 7.0. The HEPES buffer (50 mM, pH 7.0) was the ideal adsorption buffer. The isotherm of adsorption was established to determine the capacity of adsorption. The capacity of adsorption was 1.38 mg/g, and the adsorption isotherm fitted the Freundlich equation well with a correlation coefficient (R^2^) of 0.96967. Histidine, phenylalanine, cysteine, and phosphate could affect the adsorption obviously. The adsorption mechanism suggested that the forces between dextranase and Mg/Fe-LDH are electrostatic and hydrophobic. The six-histidine tags dextranase from engineered *E. coli* BL21 (DE3) KQ11-Cold III could significantly affect the adsorption. The biohybrid material of Mg/Fe-LDH, Mg/Fe-LDH/dextranase was characterized using XRD and FTIR. The XRD patterns showed that the average interlayer distance between materials was d(LDH) = 7.9934 Å and d(LDH/dextranase) = 8.1551 Å. The adsorption occurred on the surface of the LDH. The LDH morphology of the hexagonal was not affected after adsorption. The FTIR patterns confirmed the immobilization of dextranase onto LDH. The TEM images also showed that the structures of Mg/Fe-LDH did not change after adsorption of the dextranase.

## Supplementary Materials

The following are available online at http://www.mdpi.com/2079-4991/8/3/173/s1, Figure S1: Effect of different buffers on the adsorption of dextranase onto Mg/Fe-LDH, Figure S2: Modeling according to Langmuir equation, Figure S3: Effects of arginine on the adsorption(A) and elution of dextranase(B), Figure S4: Effects of lysine on the adsorption(A) and elution of dextranase(B), Table S1: The classification of amino acids in the protein sequence of dextranase.
