# Supplementary material for: The Adsorption of Dextranase onto Mg/Fe-Layered Double Hydroxide: Insight into the Immobilization
Source: Nanomaterials (Basel). 2018 Mar 19;8(3):173. doi: 10.3390/nano8030173 (PMC5869664; doi:10.3390/nano8030173)
Supplement: Supplementary file 1 [file nanomaterials-08-00173-s001.pdf]

Supplementary materials:

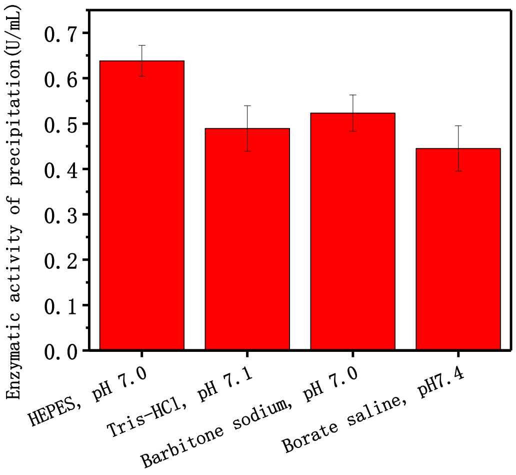

Fig. S1 Effect of different buffers on the adsorption of dextranase onto Mg/Fe-LDH

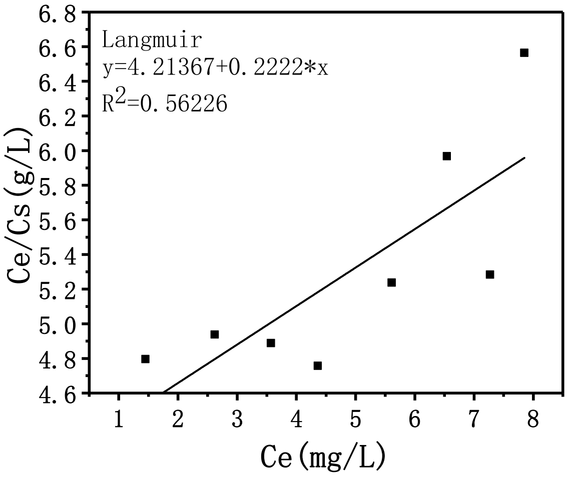

Fig. S2 Modeling according to Langmuir equation

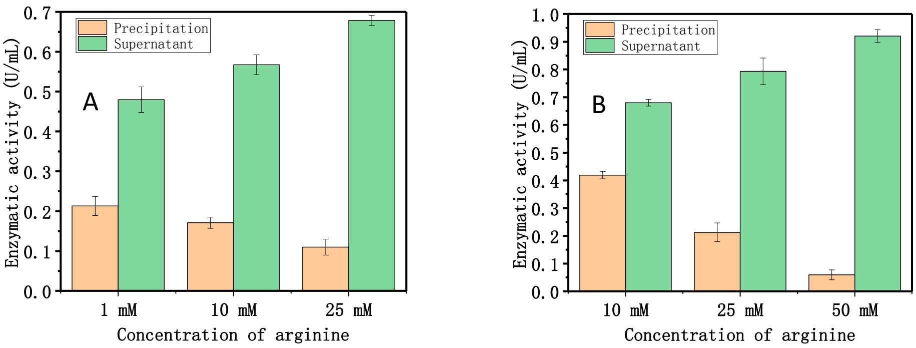

Fig. S3 Effects of arginine on the adsorption(A) and elution of dextranase(B)

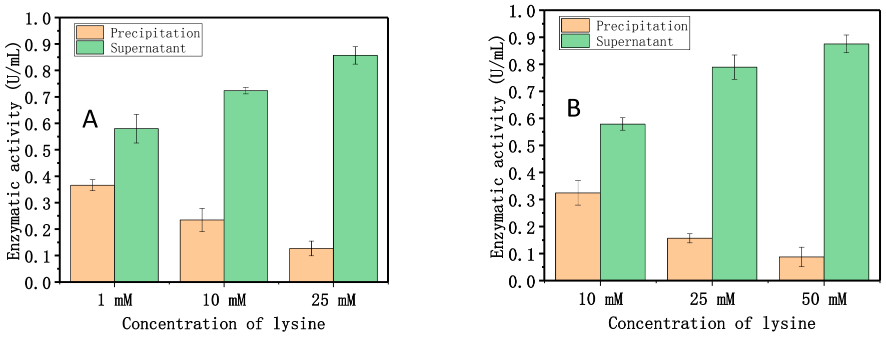

Fig. S4 Effects of lysine on the adsorption(A) and elution of dextranase(B)

The sequence of amino acids of dextranase from *A. Oxydans* KQ11:

MPGTGLGRLAKRMTAAAAVFLISSGAVLPAQAATTAGHTPSTAPAAPTDKHAITTADNGNLH
TWWHDNAVFNTTGPTGNDEVRRSSFYDLQVAQENQPDKAYDAFTYMSIPRSGKDKIGYTKED
GAEFSSQAGLTMSWSSFEYAKDVWVDVSLRTGQTITSADQVQIRPSSYNFEKQLVDADTVKIKVP
YSDAGYRFSVEFEPQLYTAYNDMSGDSGKLTTEAEGNRAIHTEPRNSMMIFAEPKLRGEQKERL
VPTEESGSIHYPAEGEVTNLNAVTEEIIYFKPGTYSMGSDYHAVLPPNVKWVYLAPGAYVKGAF
RFLHDNQSQYKVTGYGVLSGEQYVYEADTNNNYNHLSGASNCHSSCVKMLQFASADAEQKLD
LQGVTVAEPPYHSFVVYGNEQTFHMNVENYKQVGSWYWQTDGIELYKGSTMKNTFFNANDD
VLKMYHSDVTIDNTVIWKNENGPVIQWGWTPRNIDNVNVTNTTVIHNRMYWKDVKYNTCIL
NSSSHWEDMGSTTKADPNTTVKNMRFENITVEGMTNCAIRVYALSDTENIHVKNLNIDAWNG
LDWTSQVSHLKRYTNPAGEKVTIGNEIPDGNGLALENYSVGGEVIEKTADNWADHQLGRIGFD
GENWNSWNAWRTTPQ

Table S1 The classification of amino acids in the protein sequence of dextranase

Name	Number	Classification
Histidine	16	Cationic AAs
Arginine	17	Cationic AAs
Lysine	30	Cationic AAs
Alanine	51	Hydrophobic AAs
Methionine	16	Hydrophobic AAs
Leucine	32	Hydrophobic AAs
Proline	27	Hydrophobic AAs
Valine	45	Hydrophobic AAs
Tyrosine	32	Hydrophobic AAs
Phenylalaine	30	Hydrophobic AAs
Isoleucine	27	Hydrophobic AAs
Asparagine	51	Hydrophobic AAs
Tryptophane	18	Hydrophobic AAs
Other AAs	259	
**Total**	641	
